# Prognostic miRNA classifiers in t cell acute lymphoblastic leukemia: Study protocol for a systematic review and meta-analysis of observational clinical studies

**DOI:** 10.1097/MD.0000000000014569

**Published:** 2019-03-01

**Authors:** Shanthi Sabarimurugan, Madhav Madurantakam Royam, Chellan Kumarasamy, Gothandam Kodiveri Muthukaliannan, Suja Samiappan, Rama Jayaraj

**Affiliations:** a School of Biosciences and Technology, Vellore Institute of Technology (VIT), Vellore, India; b University of Adelaide, North Terrace Campus, Adelaide, Australia; c Department of Biochemistry, Bharathiar University, Coimbatore, Tamil Nadu, India; d College of Health and Human Sciences, Charles Darwin University, Casuarina, Northern Territory, Australia.

**Keywords:** biomarker, hazard ratio, meta-analysis, miRNA, patients clinical outcomes, patients survival, prognosis and T-ALL, publication bias, systematic review

## Abstract

**Background:**

The prognostic value of microRNA (miRNA) expression in T-cell acute lymphoblastic leukemia (T-ALL) has generated significant research interest in recent years. However, most diagnostic and prognostic studies with regards to miRNA expression have been focused on combined B cell and T cell lymphoblastic leukemia. There are very few studies reporting the prognostic effects of miRNA expression on T-ALL. Therefore, a pioneer systematic review and meta-analysis was proposed to explore the possibilities of miRNAs as viable prognostic markers in T-ALL. This study is intended to be useful as a guideline for future research into drug evaluation and targeting miRNA as a biomarker for the treatment and prognosis of T-ALL.

**Methods:**

The systematic review will be reported according to the Preferred Reporting Items for Systematic Review and Meta-Analysis (PRISMA) guidelines. The study search will be conducted by using Cochrane, EMBASE, Medline, Science Direct, and SCOPUS bibliographic databases. The reference lists of included studies will be manually searched to further bolster the search results. A combination of keywords will be used to search the databases.

**Discussion:**

To explore the effect of miRNA on prognosis, forest plots will be generated to assess pooled HR and 95% CI. Upregulation, downregulation, and deregulation of specific miRNAs will be individually noted and used to extrapolate patient prognosis when associated with risk factors involved in T-ALL. Subgroup analysis will be carried out to analyze the effect of deregulation of miRNA expression on patient prognosis. A fixed or random-effects model of meta-analysis will be used depending upon between-study heterogeneity. This systematic review and meta-analysis will identify and synthesize evidence to determine the prognosis of miRNA in T-ALL and suggest the possible miRNA from meta-analysis results to predict as a biomarker for further detection and treatment of T-ALL.

## 1 Introduction

### 1.1 Background

Lymphoblastic leukemia is a heterogeneous and diversified group of cancers, characterized by abnormal growth and transformation of blood cells progenitors during their differentiation in bone marrow. There are 2 major types of lymphoblastic leukemia called acute lymphoblastic leukemia (ALL) and chronic lymphoblastic leukemia (CLL). ALL is the most common malignancy in childhood cases of cancer, with B-cell lineage ALL having an incidence rate of 85% and T-cell lineage having an incidence rate of 15%.^[1]^ With regards to ETP (early T-Cell precursor) subtype prediction, 15% of all T-cell acute lymphoblastic leukemia (T-ALL) cases is associated with treatment failure.^[2]^ It is a frequently fatal hematological malignant disorder that originates in a single B or T cell lymphocyte progenitor.^[3]^ Most of the ALL cases are B-lineage ALL which represents 85% of childhood and 75% of all adult ALL cases^[4,5]^ while T-cell ALL is diagnosed in approximately 10% to 15% of children and 25% of all adults affected with ALL.^[6,7]^

### 1.2 Epidemiology

United States of America (USA) Surveillance, Epidemiology and End Results (SEER) 2017 cancer registries estimated 5970 (0.4%) new cases of T-ALL out of which 1,440 (0.2%) cases were reported as leading to death.^[8]^ A study conducted by Wang and Vose in 2013 mentioned a decline in the incidence rates of many B-cell lymphomas in the USA. In contrast, the incidence rates of T-cell lymphomas have continued to rise.^[9]^ T-ALL represents 10% to 15% of pediatric and 20% to 25% of adult cases of ALL in Europe, the United States, and Japan^[10,11]^ and is twice as prevalent in males when compared to females.^[12]^ African–American and Hispanic individuals have also been observed to have lower survival rates than Caucasian and Asian individuals. However, poor access to treatment may underlie this ethnic/regional discrepancy seen with regards to survival rates in ALL.^[13]^

MicroRNA (miRNAs) are a class of highly conserved noncoding RNAs with a length of 22 nucleotides that participate in a wide range of biological processes such as proliferation, differentiation, and apoptosis.^[14]^ Anomalous and abnormal expression of miRNAs has been increasingly recognized influencing progression in various types of tumors including lymphomas.^[15]^ About half of all human miRNAs are located at cancer-related regions of the genome and have been reported to act as oncogenes or tumor suppressor genes, implying their essential roles in progression and prognosis of cancer including, ALL.^[16,17]^ There has been a recent increase in the number of studies investigating the relationship between cancers and miRNAs in recent years, but comprehensive research and scientific literature regarding miRNAs in ALL are scarce.^[18]^

### 1.3 Rationale

#### 1.3.1 The importance of the issue

Several studies have previously described the role of miRNAs in malignant T-cell transformation and have identified both, tumor-promoting (*miR-19b*, *mir-20a*, *miR-26a*, *miR-92,* and *miR-223*)^[19]^ as well as a tumor suppressing (miR-150, *miR-155*, *miR-200,* and *miR-193b-3p*)^[20,21]^ miRNAs in T-All patients. The miRNA intracellular regulation is intricately involved in T-ALL disease biology. Although this neoplastic disorder originates from the thymus, it rapidly spreads throughout the system and is frequently fatal without therapy, when compared to the common B cell lineage ALL. Hence the proposed study will focus on T-ALL alone. There is a lacuna on how miRNAs contribute to the prognosis of T-ALL when compared to their contribution to the prognosis in B-cell lymphoblastic leukemia (B-ALL), and hence, this study focused on quantifying the effect size measures from the patient's survival through meta-analysis to better understand the survival outcome.

#### 1.3.2 How will the study address the issue?

The collective data from previously published studies, as analyzed in this systematic review and meta-analysis will help us to understand the significant role of miRNA expression in T-ALL patients. T-ALL has generally been associated with poor patient prognosis.^[12]^ The current study focuses on both up-regulated and down-regulated miRNAs individually, which provides clarity regarding the predictive power of miRNA as a biomarker for diagnosis and prognosis in T-ALL patients. Subgroup analysis based on demographic factors will help us to know more about the effect size of survival outcome in T-ALL patients. The clinical utility of miRNAs has not been established yet, and therefore this study has the potential to develop the significance of miRNAs as biomarkers in T-ALL patients. This is the first and foremost proposed study on systematic review and meta-analysis study in miRNA expression on survival of T-ALL patients.

#### 1.3.3 How will it help?

This study should also highlight significantly expressed miRNAs, providing potential miRNA targets in T-ALL, thereby benefiting researchers who wish to pursue future research in this field, as well as clinicians when estimating T-ALL patient prognosis. The study intends to provide more knowledge on the probable survival outcome in T-All patients by using pooled hazard ratio (HR) and 95% confidence interval (CI). This approach may also aid in the individualized treatment of T-ALL patients.

### 1.4 Review questions

The objective of our systematic review protocol is to describe the methodological approach for conducting a systematic review and meta-analysis to explore the viability of miRNAs as a viable prognostic marker in T-ALL.

1. Does miRNA expressions affect T-ALL patients survival?
2. What is the significance of upregulated and downregulated miRNA's expression on T-ALL patient's survival?
3. How much does the effect size of survival outcomes (HR) of T-ALL patients vary across studies?
4. Does the effect size of survival outcomes (HR) of T-ALL patients vary by subgroups?

## 2 Materials and methods

This study aims to evaluate the prognostic effect of miRNA expression in T-ALL patients. The research protocol follows the Preferred Reporting Items for Systematic Reviews and Meta-Analysis Protocol (PRISMA-P), 2015 guidelines for systematic review and meta-analysis.^[22]^

**PROSPERO registration number**: The study was registered in PROSPERO and has been assigned the registration ID: CRD42017079090

### 2.1 Search methods for identification of studies

The search for studies will be performed by utilizing online bibliographical databases including Cochrane, EMBASE, MEDLINE, SCOPUS, and Web of science will be searched until December 2018, to identify all the articles that evaluate the prognostic accuracy of miRNAs in T-ALL, a predefined search strategy outlining and combining the following terms and keywords will be used. The proposed database search using the medical subjective headings (MeSH) search terms have been included in Table 1. Relevant studies will also be collected from the reference lists of screened articles, to avoid missing any eligible study. Certain keywords will also contain subsets for better accuracy during the search.

**Table 1:**
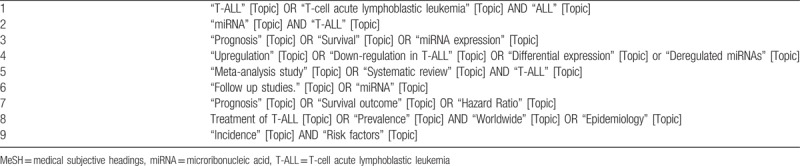
The MeSH search terms utilized in the search strategy.

### 2.2 Study selection

**Figure 1:**
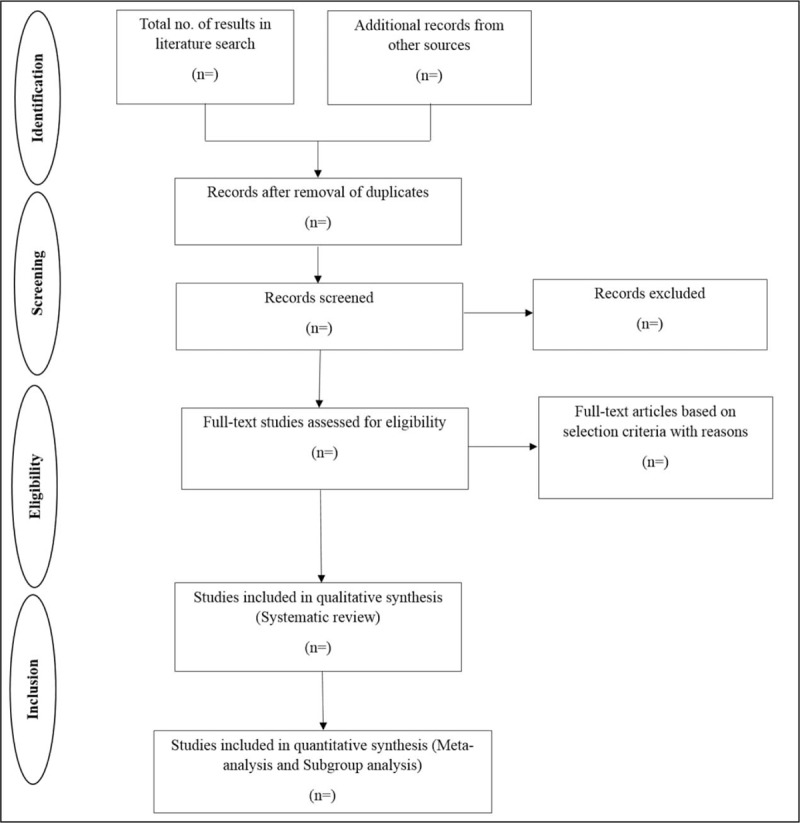
Schematic representation of the selected articles.

Studies screened from the online bibliographical databases will be from 2010 to 2018. Following the search, all identified citations will be collated and uploaded into EndNote^TM^ (Clarivate Analytics, PA) and duplicates will be removed. Titles and abstracts will then be screened by 2 independent reviewers for assessment against the inclusion criteria for the review. The full text of selected citations will be retrieved and assessed in detail against the inclusion criteria by 2 independent reviewers. Full-text studies that do not meet the inclusion criteria will be excluded, and reasons for exclusion will be provided in an appendix in the final systematic review report. Included studies will undergo a process of critical appraisal. The results of the search will be reported in full in the final report and presented in a PRISMA flow diagram and the model from PRISMA guidelines has attached as Figure 1. Any disagreements that arise between the reviewers will be resolved through discussion or with a third reviewer.

### 2.3 Selection criteria

The studies fulfilling the following criteria will be included in the systematic review.

#### 2.3.1 Inclusion criteria

1. Studies that report the miRNA expression from T-lineage cells.
2. Sample size, follow-up period, miRNA expression analysis, HR and associated 95% CI or Kaplan–Meier (KM) curve are required for the eligible studies.
3. Studies that discuss upregulation, downregulation or deregulation of miRNA expression in T-ALL patients.
4. Studies that are appropriate to PRISMA guidelines for systematic review and meta-analysis.

#### 2.3.2 Exclusion criteria

1. Letters to the editors, case reports, case studies, fact sheets, conference abstracts and review articles of T-ALL will be excluded.
2. Unpublished studies and non-English articles will not be included.
3. In vitro and animal studies on miRNA expression will not be included.
4. Studies investigating a patient/population cohort that has been represented previously in another study will be excluded.

### 2.4 Assessment of methodological quality

Selected studies will be critically appraised by 2 independent reviewers at the study level for methodological quality using quality assessment template based on the National Heart, Lung and Blood Institute (NHLBI) for Observational cohort and cross-sectional studies.^[23]^ This tool has also adopted from the prior published article.^[24,25]^ Any disagreements between the reviewers, during the appraisal, will be resolved by the involvement of a third reviewer. This assessment template will be used to evaluate the selected full-text studies which will be considered eligible for systematic review (rated as good, fair and poor) (Table 2). There are 14 elements to be analyzed and to be rated. Sample size, study period, follow up period, survival outcome, evaluation of exposure, miRNA expression, and outcome variables, statistical adjustment, and other factors will be recorded and assessed as quality appraisal elements.

**Table 2:**
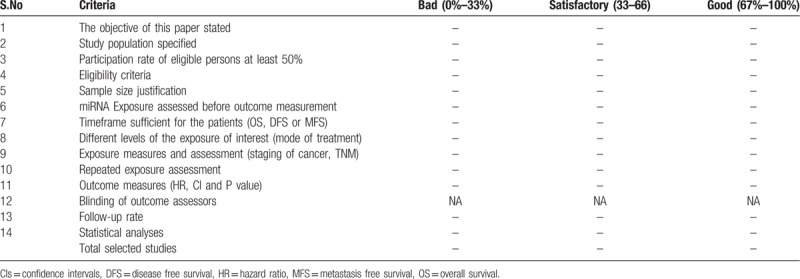
Quality assessment of the selected studies.

### 2.5 Data extraction and management

Data will be extracted from papers will be included in Microsoft Excel master sheet framework will be developed to record demographic data characters which are extracted from each qualified investigation for evaluation of study quality and data synthesis. The data extracted will include specific details about the interventions, populations, study methods and outcomes of significance to the review question and specific objectives. Any disagreements that arise between the reviewers will be resolved through discussion, or with a third reviewer. Authors of papers will be contacted to request missing or additional data where required.

The following information will be extracted from the eligible studies.

1) Author details, Publication year, the country from which patients enrolled for study, sample Size, a period of study, follow up period, sampling procedure, the source of samples, methods used for miRNA detection.
2) Demographic characteristics will be included which are age, gender details, type of lineage, the sample size for T cell lineage patients.
3) Clinicopathological characteristics of study participants which include splenomegaly, enlarged thymus gland, bleeding, bruising, recurrent infections, and hyperkalemia related symptoms will be noted from the selected studies.^[26]^ Extranodal involvement, the presence of pleural effusion, Hematoxylin and Eosin sections, pericardial effusion, and mediastinal compression symptoms (a cough or superior vena cava syndrome) will also be noted.^[27]^
4) Immunohistochemical examination like immature nature of T-cell lineage commitment, Cluster of differentiation (CD) markers such as CD3, CD 5, and CD7 will be used to predict T-cell lineage, and those data will also be extracted if the selected study has provided.^[27]^
5) Biological characteristics such as white blood cell count, lactate dehydrogenase, Bone marrow cytogenetic analysis, cytogenetic differences, immunophenotyping, karyotyping and molecular genetic analysis results will also be recorded.
6) The methodology used for the miRNA quantification along with the source of the samples, miRNA expression in T-ALL patients, up-regulated, down-regulated and deregulated miRNAs, miRNA expression rates during the follow-up period, HR estimates with 95% CI for overall survival (OS), disease-free survival (DFS), and disease-specific survival (DSS)

### 2.6 Publication bias

Publication bias will be assessed visually by the symmetry of funnel plots constructed with HR (study size in the vertical axis) and 95% CI values (effect size in the horizontal axis) which is used to observe the funnel-shaped plot. To discuss any systematic review and meta-analysis study, publication bias has to be studied and reported.^[28,29]^ The quantitative analysis of publication bias performed by utilizing the Funnel plot, “Orwin's and classic fail-safe N test”,^[30]^ “Begg and Mazumdar Rank correlation test”,^[31]^ Harbord-Egger's Test of the intercept^[32]^ and “Duval and Tweedie's trim and fill” calculation.^[33]^

#### 2.6.1 Funnel plot

The funnel plot measures, the study size, which is a mainly standard error or precision marks on the vertical axis and the function of effect size on the horizontal axis. Publication bias results in the symmetry pattern which declares presence and absence of bias exist by demonstrating the study distribution in the funnel.

#### 2.6.2 Classic fail-safe N and the Orwin fail-safe N

These 2 tests of fail-safe N will be used to determine the results which are missing from the systematic review and meta-analysis by online bibliographical database searching. Both tests will be applied to estimate studies that are missing from systematic review and meta-analysis.

#### 2.6.3 Begg and Mazumdar Rank correlation test

Begg and Mazumdar Rank correlation test Kendall's tau rank correlation between the standardized effect sizes and standard errors (which is driven primarily by sample size) by interpretation of value zero. Positive values of the test indicate a high level of accuracy among included studies, regardless of minimal sample sizes.

#### 2.6.4 Egger's test of the intercept

Egger suggests assessment of bias by using precision which is the inverse of the standard error to predict the standardized effect which means, the effect size divided by the standard error and determines the accuracy of the included study. Hence, in this equation, the size of the prognostic effect will be captured by the slope of the regression line (B1) while the intercept (B0) captures bias.

#### 2.6.5 Duval and Tweedie's trim and fill method

This study in the publication bias sector will be performed if the funnel plot appears asymmetry in which means to remove the most extreme small sides on the positive side and to recompute the effect size to each iteration until the plot appears symmetry in visually. Hence it is known as ’Trim and Fill’ where it initially trims the studies which are asymmetric from the right-hand side of the plot to locate the unbiased effect. And finally, it fills the plot by re-inserting the trimmed studies on the right along with their imputed counterparts to the left of the mean effect.

### 2.7 Data synthesis

All included studies will be reviewed and analyzed in 2 separate steps. The first step will involve the identification and dissemination of all data sources collected, followed by data extraction. The second step will involve utilization of the extracted data for statistical analysis, to estimate the pooled HR and 95% CI of patient survival in T-ALL patients using Comprehensive Meta-Analysis (CMA) software (version 3.3.070) to perform a subsequent meta-analysis. The list of significant miRNAs will be categorized based on *P* values and mean fold change. The mean fold of risk categories of miRNAs will be tabulated. HR from various endpoints (OS, DSS, DFS, and leukemia-free survival [LFS]) will be adopted for prognostic evaluation in the current meta-analysis. The mean effect of HR will be used in addition to the statistical significance of the parameter and sample size in the included studies.^[34]^

### 2.8 Assesment of heterogeneity

To determine the heterogeneity of combined HRs, Cochran's Q test and Higgins’ (I^2^) statistic will be used. Heterogeneity between the studies will be assessed using the I^2^ statistic, where an I^2^ value greater than 50% is considered indicative of substantial heterogeneity.^[35,36]^ Depending upon the heterogeneity, a random or fixed effects model will be applied to the meta-analysis. A *P* value of <.01 is to be considered statistically significant for the Q test. A random effect model will be applied if heterogeneity is observed and tau-squared statistics will be used to estimate the variation of heterogeneity between the test accuracy observed in different studies.^[37]^ The z-test will also be included in the meta-analysis to indicate the number of standard deviations from the study mean that each study may deviate. The Q statistic will be used to study the null hypothesis and to quantify the depression; the tau squared will be used. The variance between the studies will be reflected in the value of tau squared.^[38,39]^ Forest plots will be built utilizing subsets from these aggregate examinations and will likewise be utilized for subgroup analysis for individual miRNA.

### 2.9 Subgroup analysis

Subgroup analysis will be considered based on the characteristics which may offer a better resolution into the outcome effects observed in the primary meta-analysis.^[40]^ Age, gender, miRNAs, and risk factors are the tentative groups for subgroup analysis. The data and survival analysis from demographical characters, clinicopathological and biological factors of the available data from eligible studies will be considered for subgroup analysis. CD markers, immunohistochemical detection data, the risk of recurrence will also be considered as additional parameters for subgroup analysis. Furthermore, miRNA subgroup analysis will be done based on the repeated miRNAs study from a selected pool of studies. The heterogeneity of relative contributions of the study designs, populations, and periods associations with 1 or more study key variable will be assessed using meta-regression analysis. The impact of proportional contributions of these variables individually and in combination on fitting co-variables including gender distribution, methods of data collection, sample size, research quality, and sampling procedure will be calculated using meta-regression model. Tables, flowchart and figures will be plotted to depict the results in an informative and appealing manner.

### 2.10 Assessing certainty in the findings

The findings in the systematic review and meta-analysis will be summarised in a flow diagram^[41]^ and PRISMA-P checklist^[22]^ that will outline the selection process as per PRISMA guidelines (2015 Statement) for reporting systematic reviews and meta-analysis.^[22]^ The Summary of Findings will present the following information where appropriate: effect size of miRNA expression and its survival outcome in T-ALL patients vary across the studies and among subgroup analysis, overall significance of upregulated and downregulated miRNA expression and publication bias.

## 3 Discussion

In this study, the prognostic value of miRNAs as biomarkers of T-ALL will be evaluated based on observations made in relevant previous studies. Since there is comparatively minimal knowledge on T-ALL when compared to B-ALL, the present study is planned to produce better outcome measures, which help future researchers to identify new drug targets and treatment modalities. Data procured from T-ALL patient samples will be studied in this systematic review and meta-analysis. Apart from the prognostic evaluation, this study will also discuss the risk factors associated with miRNA complications in T-ALL. The various miRNAs will be discussed in the meta-analysis study based on their statistical interpretation. The specificity and sensitivity of different miRNAs will be studied under subgroup analysis. The regulation of miRNAs including upregulation, downregulation, and deregulation of miRNA will be reported collectively. This systematic review and meta-analysis will inform other researchers and readers to expand this research into meta-regressions and meta-signature evaluations further and has the potential to be used for treatment and management of T-ALL patients. This systematic review may guide future health policy, clinical practice, and medical research by providing information on the prognosis status on T-ALL

### 3.1 Strengths and limitations of this study

- The protocol follows the PRISMA-P guidelines.
- This study is unique in its attempt to elucidate the prognostic effects of miRNA specifically in T-ALL, as all previous collative studies have focused on combined B-cell and T-cell ALL.
- As the literature on only T-ALL is sparse, the resulting systematic review and meta-analysis are limited in its applicability in all patient scenarios. We suggest an updated systematic review and meta-analysis, a few years in the future after more data has been published to bolster the results achieved in this study.

## Acknowledgment

We would like to acknowledge the Meta-analysis concepts and applications workshop manual by Michael Borenstein for his guidelines on reporting Meta-analysis, subgroup analysis and publication bias (www.meta-analysis-workshops.com).

## Author contributions

RJ conceived this study and provided supervision and mentorship to SS. RJ and SS led the development of the study protocol and design, wrote the first draft of the protocol, and coordinated and integrated comments from co-authors RJ, CK, MRM, SS, and KMG. SS, CK, RJ, KMG, SS, and MRM critically revised and edited successive drafts of the manuscript and gave input to the final draft of the protocol. RJ provided methodological guidance on the overall development of the protocol. All authors read, refined and approved the final version of the manuscript.

**Conceptualization:** Rama Jayaraj.

**Data curation:** Shanthi Sabarimurugan, Rama Jayaraj.

**Formal analysis:** Shanthi Sabarimurugan, Rama Jayaraj.

**Investigation:** Shanthi Sabarimurugan, Madhav Madurantakam Royam, Chellan Kumarasamy, Gothandam Kodiveri Muthukaliannan, Suja Samiappan.

**Methodology:** Shanthi Sabarimurugan, Madhav Madurantakam Royam, Chellan Kumarasamy, Gothandam Kodiveri Muthukaliannan, Suja Samiappan, Rama Jayaraj.

**Project administration:** Shanthi Sabarimurugan, Rama Jayaraj.

**Resources:** Rama Jayaraj.

**Software:** Rama Jayaraj.

**Supervision:** Rama Jayaraj.

**Validation:** Rama Jayaraj.

**Writing – original draft:** Shanthi Sabarimurugan, Madhav Madurantakam Royam, Chellan Kumarasamy, Gothandam Kodiveri Muthukaliannan, Suja Samiappan, Rama Jayaraj.

**Writing – review & editing:** Shanthi Sabarimurugan, Madhav Madurantakam Royam, Chellan Kumarasamy, Gothandam Kodiveri Muthukaliannan, Suja Samiappan, Rama Jayaraj.

Rama Jayaraj orcid: 0000-0002-2179-0510.

## References

[1] Pui CH Robison LL Look AT Acute lymphoblastic leukaemia. Lancet (Lond, Engl) 2008;371:1030–43. 10.1016/S0140-6736(08)60457-2 18358930

[2] Coustan-Smith E Mullighan CG Onciu M Early T-cell precursor leukaemia: a subtype of very high-risk acute lymphoblastic leukaemia. Lancet Oncol 2009;10:147–56. 19147408 10.1016/S1470-2045(08)70314-0 PMC2840241

[3] Bhatia S Kaul D Varma N Functional genomics of tumor suppressor miR-196b in T-cell acute lymphoblastic leukemia. Mol Cell Biochem 2011;346:103–16. 20924650 10.1007/s11010-010-0597-0

[4] Pui CH Relling MV Downing JR Acute lymphoblastic leukemia. New Engl J Med 2004;350:1535–48. 15071128 10.1056/NEJMra023001

[5] Pui CH Jeha S New therapeutic strategies for the treatment of acute lymphoblastic leukaemia. Nat Rev Drug Discov 2007;6:149–65. 17268486 10.1038/nrd2240

[6] Baak U Gokbuget N Orawa H Thymic adult T-cell acute lymphoblastic leukemia stratified in standard- and high-risk group by aberrant HOX11L2 expression: experience of the German multicenter ALL study group. Leukemia 2008;22:1154–60. 18368072 10.1038/leu.2008.52

[7] Copelan EA McGuire EA The biology and treatment of acute lymphoblastic leukemia in adults. Blood 1995;85:1151–68. 7858247

[8] Institute NC. Cancer Stat Facts: Leukemia—Acute Lymphocytic Leukemia (ALL). 2015 Available at https://seer.cancer.gov/statfacts/html/alyl.html [access date December 21, 2018].

[9] Wang SS Vose JM Epidemiology and prognosis of T-cell lymphoma. T-cell Lymphomas 2013;Totowa, NJ: Humana Press, 25-39.

[10] Hunger SP Lu X Devidas M Improved survival for children and adolescents with acute lymphoblastic leukemia between 1990 and 2005: a report from the children's oncology group. J Clin Oncol Off J Am Soc Clin Oncol 2012;30:1663–9. 10.1200/JCO.2011.37.8018 PMC3383113 22412151

[11] Litzow MR Ferrando AA How I treat T-cell acute lymphoblastic leukemia in adults. Blood 2015;126:833–41. 25966987 10.1182/blood-2014-10-551895

[12] Goldberg JM Silverman LB Levy DE Childhood T-cell acute lymphoblastic leukemia: the Dana-Farber Cancer Institute acute lymphoblastic leukemia consortium experience. J Clin Oncol Off J Am Soc Clin Oncol 2003;21:3616–22. 10.1200/JCO.2003.10.116 14512392

[13] Organista-Nava J Gomez-Gomez Y Illades-Aguiar B High miR-24 expression is associated with risk of relapse and poor survival in acute leukemia. Oncol Rep 2015;33:1639–49. 25672522 10.3892/or.2015.3787 PMC4358084

[14] Bartel DP MicroRNAs: genomics, biogenesis, mechanism, and function. Cell 2004;116:281–97. 14744438 10.1016/s0092-8674(04)00045-5

[15] Husby S Geisler C Gronbaek K MicroRNAs in mantle cell lymphoma. Leuk Lymphoma 2013;54:1867–75. 23339447 10.3109/10428194.2013.766731

[16] Calin GA Dumitru CD Shimizu M Frequent deletions and down-regulation of micro- RNA genes miR15 and miR16 at 13q14 in chronic lymphocytic leukemia. Proc Natl Acad Sci U S A 2002;99:15524–9. 12434020 10.1073/pnas.242606799 PMC137750

[17] He L Hannon GJ MicroRNAs: small RNAs with a big role in gene regulation. Nat Rev Genet 2004;5:522–31. 15211354 10.1038/nrg1379

[18] Duyu M Durmaz B Gunduz C Prospective evaluation of whole genome microRNA expression profiling in childhood acute lymphoblastic leukemia. BioMed Res Int 2014. 10.1155/2014/967585 PMC4053274 24955371

[19] Mavrakis KJ Van Der Meulen J Wolfe AL A cooperative microRNA-tumor suppressor gene network in acute T-cell lymphoblastic leukemia (T-ALL). Nat Genet 2011;43:673–8. 21642990 10.1038/ng.858 PMC4121855

[20] Mets E Van der Meulen J Van Peer G MicroRNA-193b-3p acts as a tumor suppressor by targeting the MYB oncogene in T-cell acute lymphoblastic leukemia. Leukemia 2015;29:798. 25231743 10.1038/leu.2014.276 PMC4890642

[21] Sanghvi VR Mavrakis KJ Van der Meulen J Characterization of a set of tumor suppressor microRNAs in T cell acute lymphoblastic leukemia. Sci Signal 2014;7:ra111. 25406379 10.1126/scisignal.2005500 PMC4693296

[22] Moher D Shamseer L Clarke M Preferred reporting items for systematic review and meta-analysis protocols (PRISMA-P) 2015 statement. Syst Rev 2015;4:1. 25554246 10.1186/2046-4053-4-1 PMC4320440

[23] Services USDoHH. Study Quality Assessment Tools. Available at: https://www.nhlbi.nih.gov/health-topics/study-quality-assessment-tools [access date December 21, 2018].

[24] Sabarimurugan S Madurantakam Royam M Das A Systematic review and meta-analysis of the prognostic significance of miRNAs in melanoma patients. Mol Diagn Ther 2018;22:653–69. 30259393 10.1007/s40291-018-0357-5

[25] Kumarasamy C Devi A Jayaraj RJSR Prognostic value of microRNAs in head and neck cancers: a systematic review and meta-analysis protocol. Syst Rev 2018;7:150. 30285880 10.1186/s13643-018-0812-8 PMC6169036

[26] Al-Mashaikhi A Al Khatri Z Al Mamari S Immunophenotypic characteristics of T-acute lymphoblastic leukemia in omani patients: a correlation with demographic factors. Oman Med J 2018;33:43–7. 29467998 10.5001/omj.2018.08 PMC5798795

[27] Mathan L Ananthamurthy A Clinicopathological attributes of T-lymphoblastic lymphoma seen in a tertiary care centre. Clin Cancer Investig J 2018;7:1–8.

[28] Jayaraj R Kumarasamy C Sabarimurugan S Letter to the editor in response to the article, “the epidemiology of oral human papillomavirus infection in healthy populations: a systematic review and meta-analysis”. Oral Oncol 2018;84:121–2. 30075910 10.1016/j.oraloncology.2018.07.018

[29] Jayaraj R Kumarasamy C Ramalingam S Systematic review and meta-analysis of risk-reductive dental strategies for medication related osteonecrosis of the jaw among cancer patients: approaches and strategies. Oral Oncol 2018;86:312–3. 30262125 10.1016/j.oraloncology.2018.09.017

[30] Rosenthal RJPb The file drawer problem and tolerance for null results. Psychol Bull 1979;86:638.

[31] Begg CB Mazumdar M Operating characteristics of a rank correlation test for publication bias. Biometrics 1994;1088–101. 7786990

[32] Egger M Smith GD Schneider M Bias in meta-analysis detected by a simple, graphical test. BMJ 1997;315:629–34. 9310563 10.1136/bmj.315.7109.629 PMC2127453

[33] Duval S Tweedie RJB Trim and fill: a simple funnel-plot–based method of testing and adjusting for publication bias in meta-analysis. Biometrics 2000;56:455–63. 10877304 10.1111/j.0006-341x.2000.00455.x

[34] Jayaraj R Kumarasamy C Gothandam KJCm, research. Letter to the editor “Prognostic value of microRNAs in colorectal cancer: a meta-analysis”. Cancer Manag Res 2018;10:3501. 30271198 10.2147/CMAR.S177875 PMC6145637

[35] Higgins JP Thompson SGJSim Quantifying heterogeneity in a meta-analysis. Stat Med 2002;21:1539–58. 12111919 10.1002/sim.1186

[36] Poddar A Aranha RR K Muthukaliannan G Head and neck cancer risk factors in India: protocol for systematic review and meta-analysis. BMJ Open 2018;8:e020014. 10.1136/bmjopen-2017-020014 PMC6104749 30127047

[37] Jayaraj R Kumarasamy C Madhav MR Comment on systematic review and meta-analysis of diagnostic accuracy of miRNAs in patients with pancreatic cancer. Dis Markers 2018. 10.1155/2018/6904569 PMC6217755 30425753

[38] Jayaraj R Kumarasamy C Systematic review and meta-analysis of cancer studies evaluating diagnostic test accuracy and prognostic values: approaches to improve clinical interpretation of results. Cancer Manag Res 2018;10:4669–70. 30410400 10.2147/CMAR.S183181 PMC6199231

[39] Kim KW Lee J Choi SH Systematic review and meta-analysis of studies evaluating diagnostic test accuracy: a practical review for clinical researchers-part I. General guidance and tips. Korean J Radiol 2015;16:1175–87. 26576106 10.3348/kjr.2015.16.6.1175 PMC4644738

[40] Yu N Zhang Q Liu Q A meta-analysis: micro RNA s’ prognostic function in patients with nonsmall cell lung cancer. Cancer Med 2017;6:2098–105. 28809453 10.1002/cam4.1158 PMC5603832

[41] Liberati A Altman DG Tetzlaff J The PRISMA statement for reporting systematic reviews and meta-analyses of studies that evaluate health care interventions: explanation and elaboration. J Clin Epidemiol 2009;6:e1000100. 10.1016/j.jclinepi.2009.06.006 19631507

